# Earliest evidence of Neanderthal multifunctional bone tool production from cave lion (*Panthera spelaea*) remains

**DOI:** 10.1038/s41598-025-08588-w

**Published:** 2025-07-05

**Authors:** Grégory Abrams, Patrick Auguste, Stéphane Pirson, Isabelle De Groote, Éva Halbrucker, Kévin Di Modica, Camille Pironneau, Tristan Dedrie, Carlo Meloro, Valentin Fischer, Hervé Bocherens, Yves Vanbrabant, Fabrice Bray

**Affiliations:** 1 ArcheOs, Research Laboratory for Biological Anthropology, Ghent Archaeological Sciences Centre, Department of Archaeology, Ghent University, 9000 Ghent, Belgium; 2 Scladina Cave Archaeological Centre, Espace muséal d’Andenne, 5300 Andenne, Belgium; 3 CNRS, University Lille UMR 8198 - Evo-Eco-Paléo, 59000 Lille Cedex, France; 4 Direction Scientifique et Technique, Agence Wallonne du Patrimoine, 5100 Namur, Belgium; 5 Department of Geology (RU Geology) and European Archaeometry Centre (RU Art, Archaeology, Heritage), University of Liège, 4000 Liège, Belgium; 6 Research Centre in Evolutionary Anthropology and Palaeoecology, School of Biological and Environmental Sciences, Liverpool John Moores University, Liverpool, L3 3AF UK; 7 Prehistory Research Group, Department of Archaeology, Ghent University, 9000 Ghent, Belgium; 8 Research Foundation – Flanders (FWO), 1000 Brussels, Belgium; 9 Evolution & Diversity Dynamics Lab, UR Geology, University of Liège, 4000 Liège, Belgium; 10 Department of Geosciences, Biogeology, University of Tübingen, 72074 Tübingen, Germany; 11 Biogeology, Senckenberg Centre for Human Evolution and Paleoenvironment, 72074 Tübingen, Germany; 12 Geological Survey of Belgium, Royal Belgian Institute of Natural Sciences, 1000 Brussels, Belgium; 13 UAR 3290 - MSAP - Miniaturisation pour la Synthèse, l’Analyse et la Protéomique, CNRS, University of Lille, 59000 Lille, France

## Abstract

Throughout history, humans have had a complex relationship with lions, both reverencing and fearing them. Interactions between Neanderthals and cave lions (*Panthera spelaea*) remain poorly documented due to the scarcity of direct evidence. This study examines the selective use of cave lion bones by Neanderthals to determine whether this behaviour was driven by practical, functional, or symbolic factors, through a detailed zooarchaeological analysis. Previous studies highlighted Neanderthals’ skinning and butchering of cave lions, yet new discoveries at Scladina Cave (Belgium) offer deeper insights into this relationship. Dated to the end of the Saalian, the faunal assemblage provides the earliest evidence of bone tools crafted from cave lion remains. A tibia was deliberately processed into multifunctional tools, initially serving as an intermediate tool before being repurposed as retouchers. Proteomic analysis applied on the remains, confirmed the specific identification. These findings reveal that Neanderthals not only competed with but actively utilized cave lions for practical purposes, indicating complex ecological and behavioral interactions. Relationships between pre-humans and large predators, rooted in the Middle Pleistocene, suggest a strategic exploitation of carnivore remains. However, opportunistic procurement cannot be entirely ruled out as a potential factor influencing animal choice in tool production. The intentional transformation of lion bones into functional tools highlights Neanderthals’ cognitive skills, adaptability, and capacity for resource utilization beyond their immediate survival needs.

**Supplementary Information:**

The online version contains supplementary material available at 10.1038/s41598-025-08588-w.

## Introduction

Despite their documented coexistence spanning several hundred millennia^1,2^, direct evidence of interaction between large felids and pre-humans remains exceedingly rare. The earliest evidence of the exploitation of these carnivores predates the Neanderthals. *Panthera leo fossilis* bones discovered in Layer TD10-1 of Gran Dolina (Spain, MIS 9), bearing numerous cutmarks, suggest the manipulation of a fresh carcass, perhaps even hunted, for food^3^. Moreover, a fragmentary humerus of *Homotherium latidens*, found in Layer 13 II-4C (“Spear Horizon”) of Schöningen (Germany, MIS 9), shows typical features of a bone being used as a tool (retoucher)^4,5^. This bone was obviously fragmented while still fresh, also indicating access to a fresh carcass.

Neanderthal interactions with large carnivores have long been debated, with some studies suggesting that these hominins actively hunted or scavenged cave lions (*Panthera spelaea*). So far, the earliest indications of Neanderthal exploitation appeared on a third phalanx discovered in Einhornhöhle (MIS 7-6, Germany), likely resulting from the skinning of a cave lion^6^. Some younger European sites have yielded evidence of butchering activities on cave lions, such as those found in Bolomor Cave^7^ (MIS 5, Spain) and “Chez-Pinaud”^8^ (MIS 4, France). Excavations in Siegsdorf (MIS 3, Germany) uncovered cave lion remains, including a rib with marks interpreted as possible impacts from wooden spears, accompanied by numerous butchery marks on various anatomical segments. These findings strongly suggest that Neanderthals engaged in hunting and the exploitation of these large predators^6^.

At Scladina Cave (Belgium), recent discoveries of bone tools made from cave lion bones raise questions about their function and significance. This study aims to determine whether these modifications reflect purely utilitarian practices or if they suggest a deeper behavioural pattern and contributes to discussions on Neanderthal subsistence strategies and material selection. This research relies both on zooarchaeological and taphonomic evidence as well as analytical techniques, that are ZooMS and LC–MS/MS, which have been used to assert the species identification.

**Fig. 1:**
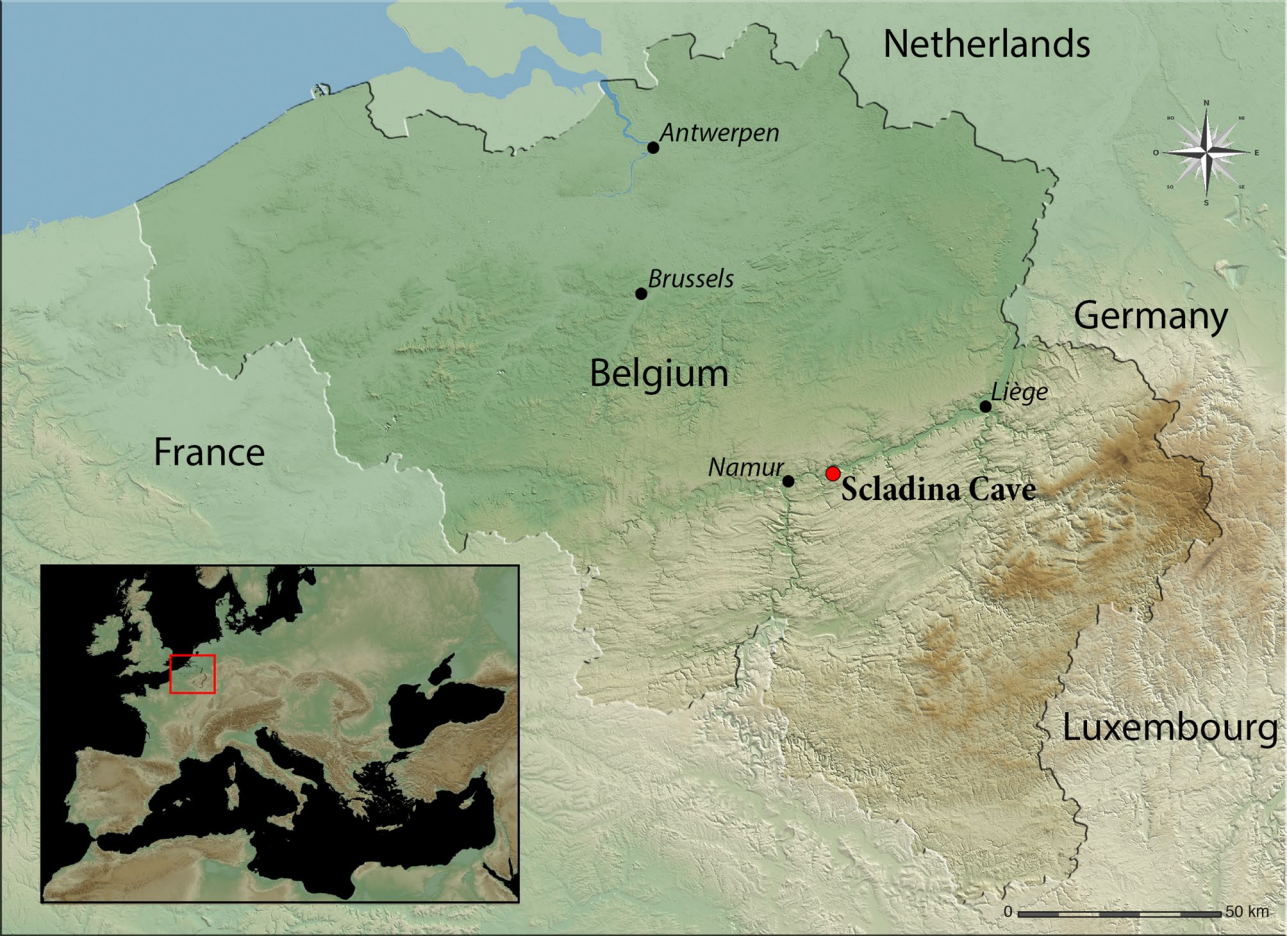
Scladina Cave is in the Meuse Valley, in Southern Belgium, close to the limit between Paleozoic limestones (Southeastern part of Belgium) and the low plateaus (Northwestern part). The cave belongs to a network connecting different caves, where Saint-Paul and Sous-Saint-Paul caves are the two other main cavities. The map was generated in Global Mapper v.22.1 using SRTM data from the NASA (https://www.earthdata.nasa.gov/data/instruments/srtm).

**Fig. 2:**
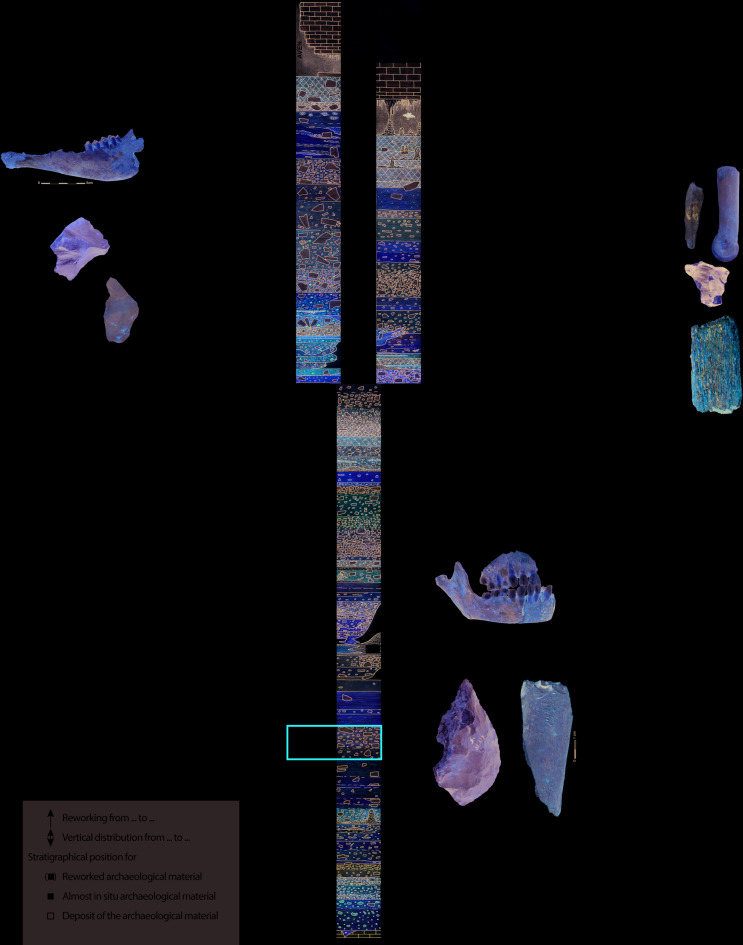
Stratigraphic sequence of Scladina Cave exhibiting the archaeological richness of the site. The sedimentary Unit 5 has yielded the main archaeological assemblage containing the bone retouchers made from carnivoran remains (*Ursus spelaeus* and *Panthera spelaea*). Above Unit 2A, the system is filled by sediments from two sources: the entrance to the cave on the right and an open doline in the ceiling of the cave on the left (Modified after Bonjean et al., 2014).

Scladina Cave, located along the right bank of the Meuse Valley between Andenne and Namur, has been under scientific investigation since 1978 (Fig. 1). The site presents an exceptionally well-preserved stratigraphic sequence, comprising at least 120 layers and spanning approximately 400,000 years (Fig. 2)^9^. Over the past two decades, extensive interdisciplinary studies have refined our understanding of the cave’s sedimentary dynamics, paleoenvironmental history, and chronostratigraphy. This robust framework contextualizes the archaeological findings and taphonomic processes affecting the site’s assemblages^10–13^.

Excavations at Scladina have yielded multiple Paleolithic assemblages, including limited Upper Paleolithic evidence associated with the Aurignacian and more substantial Neanderthal occupations linked to Mousterian industries^14–16^. The discovery of the Scladina Child, one of the best-preserved Neanderthal juvenile remains in western Europe, further highlights the site’s significance^12,13,17,18^.

The main archaeological assemblage has been retrieved within the stratigraphic Unit 5 (also referred as Vb on the terrace), which consists of a diamicton affected by solifluxion^12^. Uranium/Thorium dates recently obtained on speleothems led to position the thick CC4 speleothem from Sedimentary complex 4 in the Eemian^9^. The underlying Unit 5 is now placed at the end of the Middle Pleistocene (MIS 6, Fig. 2), consistent with the infrared stimulated luminescence on feldspar (IRSL) ages obtained from the overlying Unit 4B (153 ± 15 ka BP; Balescu in ^13^) and with the thermoluminescence (TL) date obtained on burnt flint directly from Unit 5 (130 ± 20 ka BP)^19^.

Archaeological assemblage 5 is characteristic of the Mousterian techno-complex, associated with Middle Paleolithic Neanderthal populations. The toolkit primarily comprises flint artifacts, reflecting a well-structured chaîne opératoire involving local and non-local raw material procurement, core preparation, and systematic flake production^20–22^. Retouched tools, while not abundant, include side scrapers, notches, and denticulates, often showing evidence of extended use and rejuvenation. The diversity of tool types, along with a high frequency of cortical flakes and débitage, indicates on-site tool manufacture and maintenance.

The presence of 29 bone retouchers within the archaeological assemblage 5, particularly one made from a cave bear (*Ursus spelaeus*) remains (17), points to a complex toolkit that integrated lithic and osseous technologies^23^. These tools were likely employed in retouching flint edges, as indicated by micro-chipping patterns and embedded lithic residues^23,24^. Overall, the lithic industry of Unit 5 reflects a flexible and adaptive technological behavior well-aligned with Neanderthal subsistence strategies. Zooarchaeological analyses of Unit 5 indicate that Neanderthals primarily hunted chamois (*Rupicapra rupicapra*) but also processed a wide range of species, from small mammals to megafauna^25–27^. These observations, combined with the palynological records, suggest that the Neanderthal occupation took place during a cold phase, where the environment was open; probably during a winter given the age at which the chamois have been hunted^26^.

Several carnivores have been identified within Unit 5, such as the wolf (*Canis lupus*) and the red fox (*Vulpes vulpes*), which are the most represented carnivores alongside with cave bears^23,24,28^. Among the remains, modified by Neanderthals and used as tools, are several bones belonging to the cave lion. These remains, which are quite singular, both for this period and for the animal used, are the subject of this study, which aims to determine their origin and methods of exploitation.

## Results

### Description of the bone retouchers

**Fig. 3:**
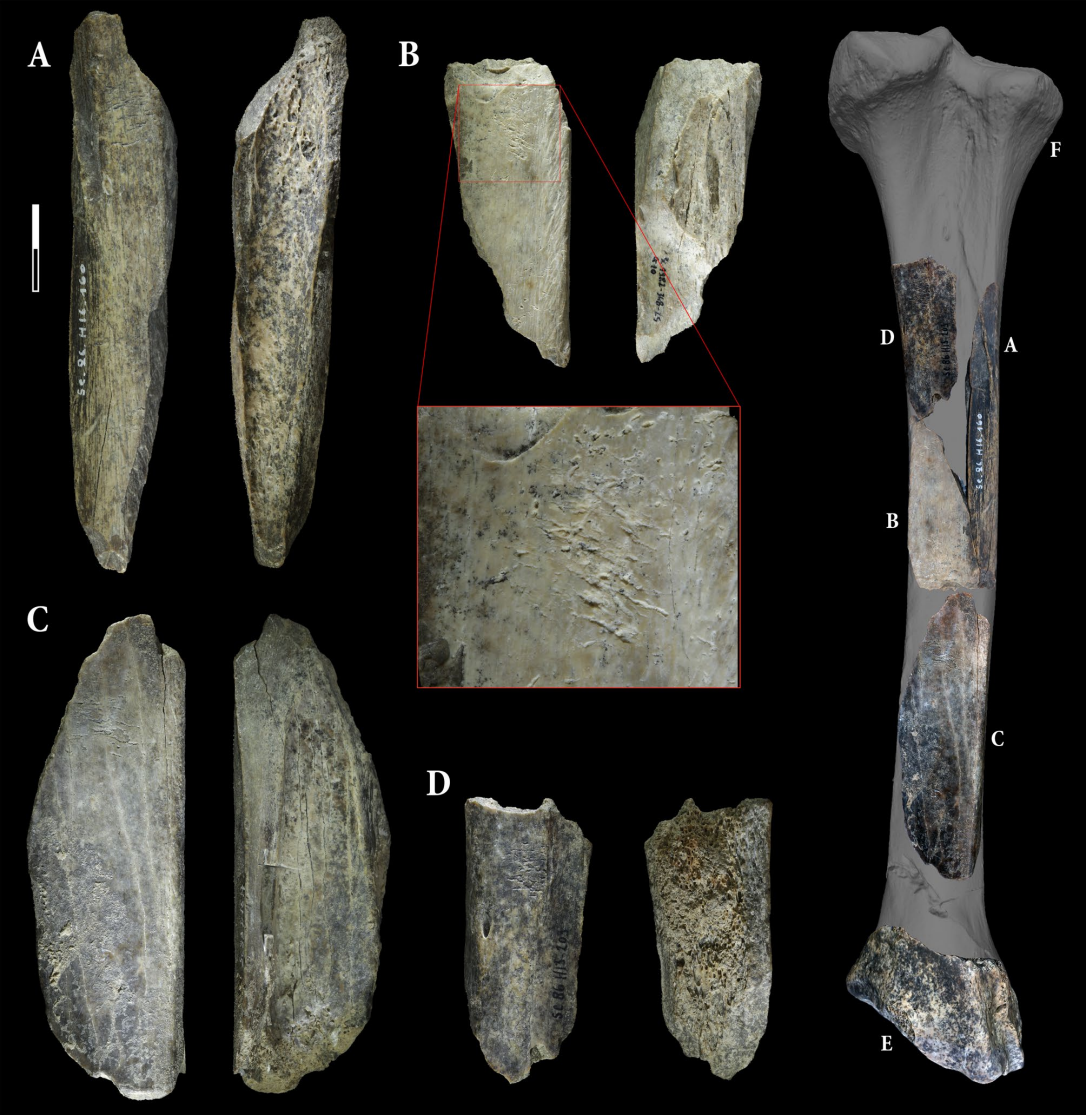
The four bone retouchers made on cave lion remains excavated in Scladina Cave Archaeological Assemblage 5—Sc1986-1278-160 (**A**), Sc1982-348-25 (**B**), Sc1986-1270-203 (**C**) and Sc1983-338-17 (**D**). Detailed picture of Sc1982-348-25 exhibits the typical pits and scores related to the use as a bone retoucher. A and B refit together (see also Fig. 5). As suggested by their relative positions projected onto a left tibia of a cave lion excavated in Goyet (**F**), the four retouchers, associated with a distal end of the tibia (**E**), seem to be crafted from a single and unique bone.

**Fig. 4:**
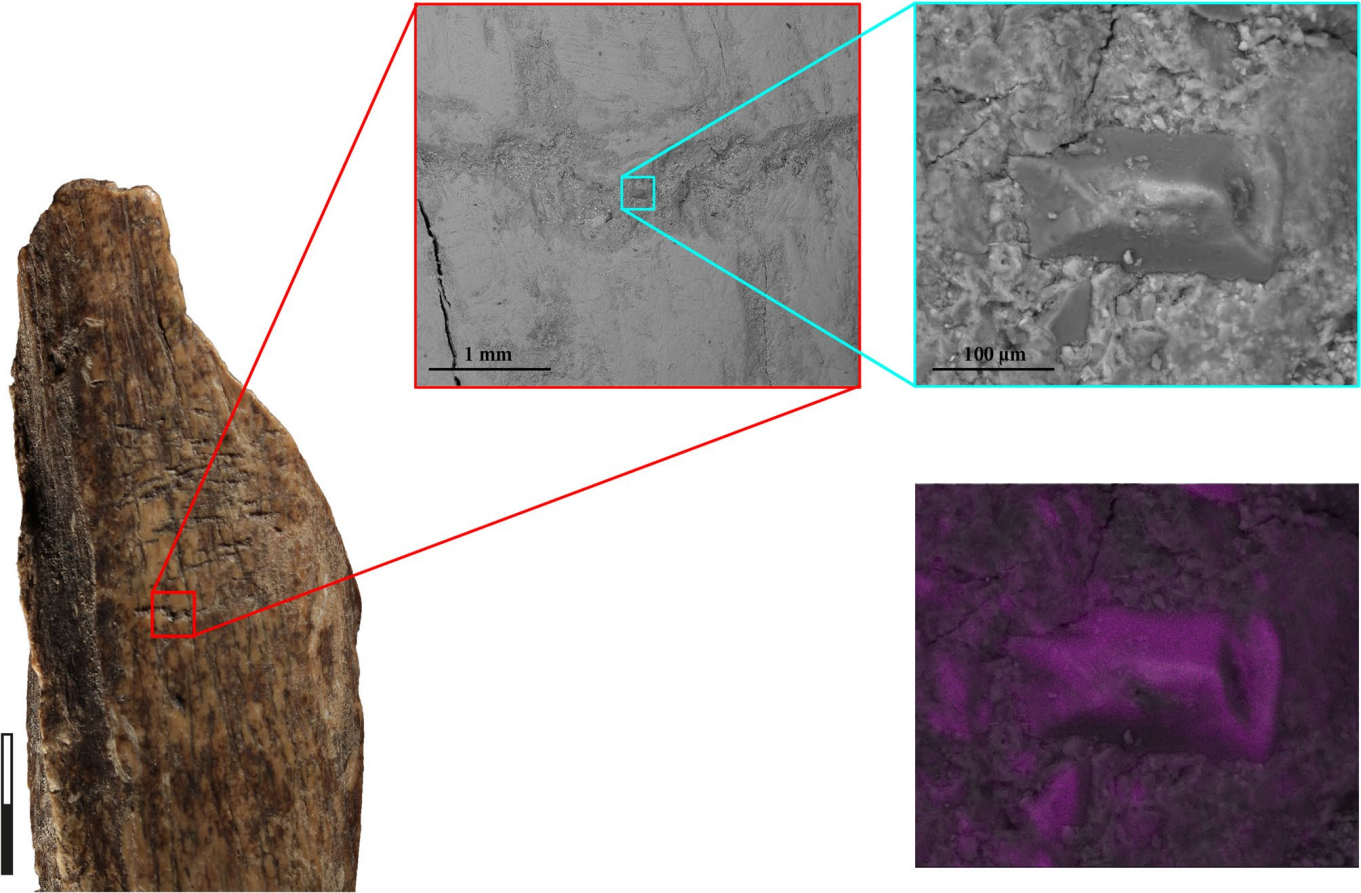
Energy-dispersive X-ray (EDX) spectroscopy analysis made on a lithic inclusion embedded in a score located in the main use area of Sc1986-1278-160. The spectroscopy highlights peaks of Si and O, which is consistent with the siliceous material. The mapping of Si highlights in purple the targeted lithic chips.

The four retouchers made from cave lion remains display typical features indicative of their use (Table 1; Fig. 3): notches, in the shape of scores and pits, are evident on the cortical surface. As per Mallye’s terminology^29^, these features are either concentrated on the primary use surfaces (Sc1982-348-25 (1), Sc1986-1278-160 (1), Sc1986-1270-203, Sc1983-338-17) or dispersed on secondary ones (Sc1982-348-25 (2), Sc1986-H16-160 (2)). The use surfaces, defined by the concentration of impact, are predominantly located in the distal part of the fragments and can be either centered or lateral as per Mallye’s terminology^29^. The observed traces on these bone tools primarily consist of rectilinear scores with smoothed-side shapes and triangular pits, suggesting they were likely used on flint^29^. Still embedded within the cortical bone, lithic chips are present in most of the bone tools. The chemical composition has been recorded for fragments embedded in Sc1986-1278-160 (Fig. 4) using Energy-dispersive X-ray spectroscopy (EDX). The recorded Silicon (Si) peak highlights the presence of exogenous siliceous materials. Unfortunately, this chemical signature does not allow us to distinguish between siliceous materials such as flint, quartz, or quartzite, which are all present in lithic materials collected within sedimentary Unit 5^20^.

**Table 1:** Description of the bone retouchers made from left tibia fragments of a cave lion (*Panthera spelaea*).

Accession number	Length (in mm)	Width (in mm)	Thickness (in mm)	N of use surface	Impact Points	Flake scars	Cut marks	Scrapping marks	Convexity	Location of the use area	Type of traces	Pits	Scores	Frequency of the scores	Use intensity	Lithic splinters
Sc1983-338-17	101	36	9	1	Yes	Yes	Yes	Yes	Plano-Convex	Lateral (left)	Scores and pits	Triangular	Rectilinear—smooth	Concentrated	+++	Yes
Sc1982-348-25 (1)	70	28	9	2	Yes	Yes	Yes	No	Plan	Centred	Scores and pits	Triangular	Rectilinear—smooth	Concentrated	+++	Yes
Sc1982-348-25 (2)										Lateral (right)		Triangular	Rectilinear—smooth	Dispersed	++	Yes
Sc1986-1278-203	65	28	8	1	No	Yes	Yes	No	Plano-Convex	Centred	Scores and pits	Triangular	Rectilinear—smooth	Concentrated	+++	No
Sc1986-1270-160 (1)	127	6	9	2	Yes	Yes	Yes	No	Plano-Convex	Centred	Scores and pits	Triangular	Rectilinear—smooth	Concentrated	++	Yes
Sc1986-1270-160 (2)						No	Yes	No	Convex	Lateral	Scores and pits	Triangular	Rectilinear—smooth	Dispersed	+	No

The frequencies of marks suggest a variable use of these tools, where one of the use surfaces (Sc1986-1278-160 (2)) appears to have not met the requested needs, as the marks are few and dispersed, unlike the other surface on the same tool that has been more intensively utilized.

The blanks exhibit typical fresh bone fracture patterns^30,31^, and this relative freshness is also evidenced by cut marks present on all blanks. Furthermore, scraping marks observed on the use area of Sc1983-338-17 suggest the removal of the periosteum prior to its use as a bone tool. The surfaces are well preserved, with minimal erosion and weathering. The spongiosa still present on Sc1986-1270-203 is also perfectly preserved. This good state of preservation allowed refitting between tools Sc1982-348-25 and Sc1986-1278-160 without significant material loss. No alterations linked to carnivore activity or advanced weathering stage was detected on the tools, nor on the other lion remains uncovered in this unit.

**Fig. 5:**
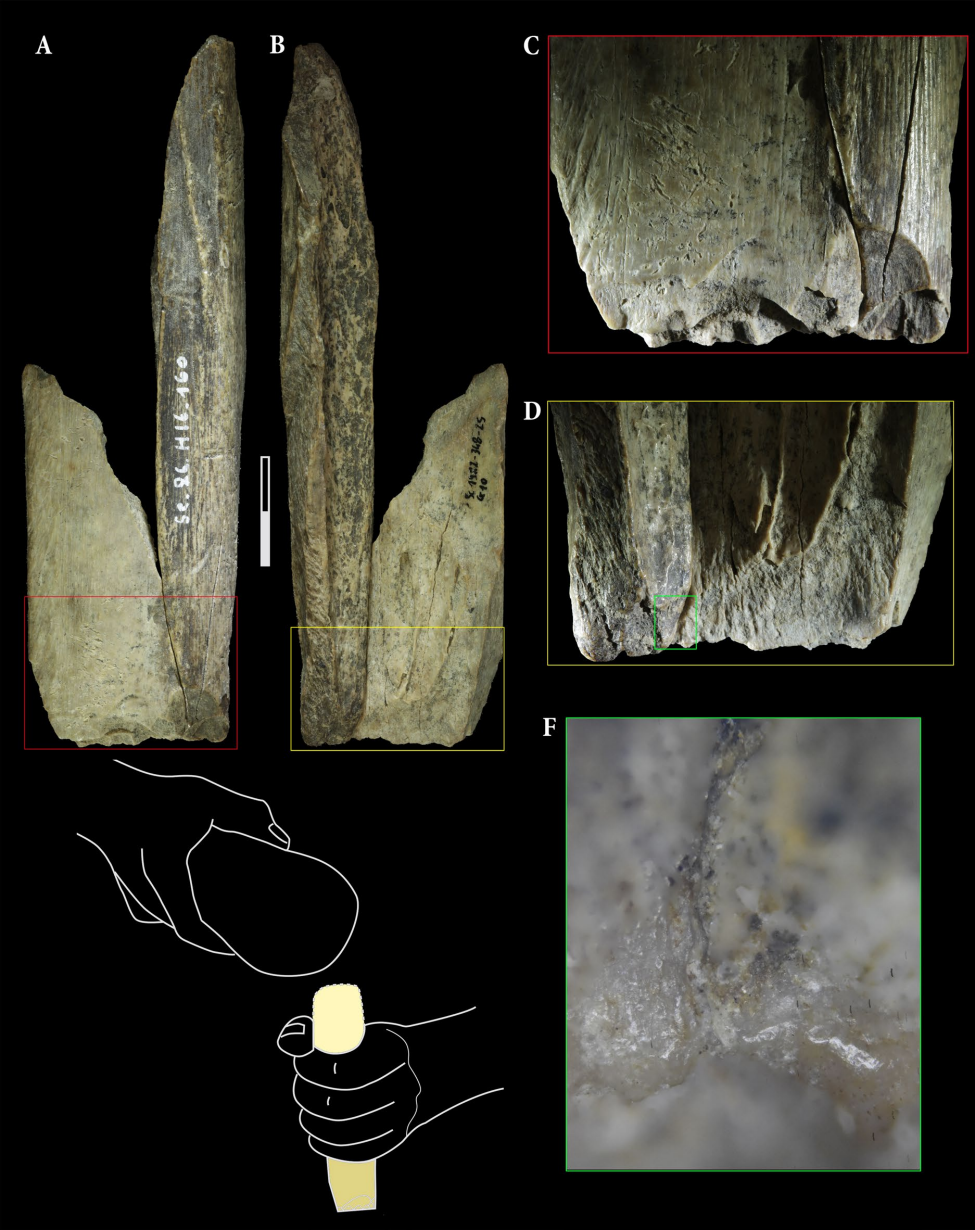
The refitting of Sc1982-345-25 and Sc1986-1278-160 allows for the reconstruction of a diaphysis fragment of a left tibia (**A**,**B**). The bifacial reshaping observed on the distal part (**C**,**D**) presents a polish (**F**) that occurred prior to the breakage of the bone as it is present on both side of the fracture. Combined with blow fractures observed on the proximal part (**A**,**B**), these are all indicators supporting the multifunctional use of this tibial fragment as an intermediate tool as suggested in the drawing by S. Lambermont (**E**), before the different fragments were used separately as retouchers as illustrated in Figs. 3A,B

Additional insights into their use are provided by impact points visible on some supports, as well as flake scars. However, among these bone tools, Sc1986-1278-160 and Sc1982-348-25 stand out (Fig. 5). Their refitting reconstitutes a fragment of left tibia diaphysis, which appears to have been carefully prepared and used (Fig. 3). After, the bone was deliberately fractured and the fragments used separately as retouchers, as previously observed with retouchers made from cave bear remains within the same context^23^. The diaphyseal tibia fragment exhibits distinct features on its apical and basal ends. The basal end has been intentionally shaped by bifacial retouching, resulting in a bevel form, contrasting with what appears to be a shattered splinter from a blow to the apical part of the blank. This bone fragment could have functioned as an intermediate bone tool, such as a chisel^32–34^. The most prominent part (Fig. 5F) shows slight polishing and chipping that do not seem related to the shaping of the piece. After closer inspection under high magnification (up to 200 x), there is some polish and rounding at the edge of the tool that is connected between the two sides of the breakage of the bone. This polish is smoother and more compact than the natural surface. The distribution of this polish is also limited to the edge of the tool, spreading a bit into the background. These observations indicate that the polish is connected to the use of the tool rather than production traces or post-depositional surface modification. From the directionality of the polish and some small edge removals that also seem connected to use, the directionality of the movement is transversal. The observed polish is smooth(ening), domed, bright, somewhat fluid, with transversal and parallel directionality, and few shallow, short, wide striations in a transversal directionality in the background and parallel on the corner of the edge. Although the shape resembles chisels previously mentioned in the literature^33^, it is still too early to assign a precise function to it. The recurring fracturing observed on long bones, particularly from chamois, to extract marrow could be one possibility, as well as the splitting of woody material along the grain. Based on the polish attributes, for now we suggest the use on bone, but further experimental and use-wear analyses are necessary for the clearer understanding of the function of this tool. In summary, the distribution, location and sequencing of the various intentional modifications—bifacial reshaping for making a chisel-like tool followed by intentional fracturing and subsequent use of the isolated fragments as bone retouchers—impart successive utilitarian functions to these artifacts.

Despite a relatively diverse faunal assemblage in the sedimentary Unit 5^26,28^, there is limited diversity in the species used for manufacturing bone retouchers. Most of these tools were made from cave bear remains (17). While some specimens have yet to be identified to the species level, the use of other taxa—such as woolly rhinoceros (*Coelodonta antiquitatis*), reindeer (*Rangifer tarandus*), horse (*Equus caballus*) or Bos/Bison (*Bos primigenius*/*Bison priscus*)—is anecdotal, as only one tool could be associated with each of these species (Table 2). The second most represented species is the cave lion, with four tools attributed to it, however, these four tools could belong to a single individual.

**Table 2:** list of retouchers and the ZooMS identification.

Accession number	Former ID	Unit	Location (Square)	ZooMS identification: Family	ZooMS identification: Genus
Sc-Ø-2647				Ursidae	*Ursus*
Sc-Ø-2648				Ursidae	*Ursus*
Sc1983-310-15	Sc83-G20-15	5	G20	Ursidae	*Ursus*
Sc1982-348-24		Vb	G10	Ursidae	*Ursus*
Sc1982-348-25		Vb	G10	Felidae	*Panthera*
Sc1982-348-26		Vb	G10	Ursidae	*Ursus*
Sc1982-348-27		Vb	G10	Cervidae	*Rangifer*
Sc1983-186-25-2	Sc83-F13-25	5	F13	Ursidae	*Ursus*
Sc1983-338-17	Sc83-D15-17	5	D15	Felidae	*Panthera*
Sc1983-382-120	Sc83-G15-120	5	G15	Bovidae	*Bos/Bison*
Sc1983-384-128	Sc83-G14-128	5	G14	Ursidae	*Ursus*
Sc1984-553-18	Sc84-614-18	5	D16	Ursidae	*Ursus*
Sc1984-614-11bis	Sc84-G16-11bis	5	G16	Ursidae	*Ursus*
Sc1984-661-97	Sc84-E16-97	5	E16	Ursidae	*Ursus*
Sc1986-1270-203	Sc86-H13-203	5	H13	Felidae/Hyaenidae	*Panthera/ Hyaenidae*
Sc1986-1278-160	Sc86-H16-160	5	H16	Felidae	*Panthera*
Sc1986-1286-185	Sc86-H16-185	5	H16	Rhinocerotidae	*Rhinoceros*
Sc1998-203-230	Sc98-B30-203	5	B30	Ursidae	*Ursus*

The table contains the accession number of retouchers, the excavation year, numbers and geological unit. The identification of family and genus was based on ZooMS results with extinct genus. ZooMS marker peptides identified in Supp. File S1.

**Fig. 6:**
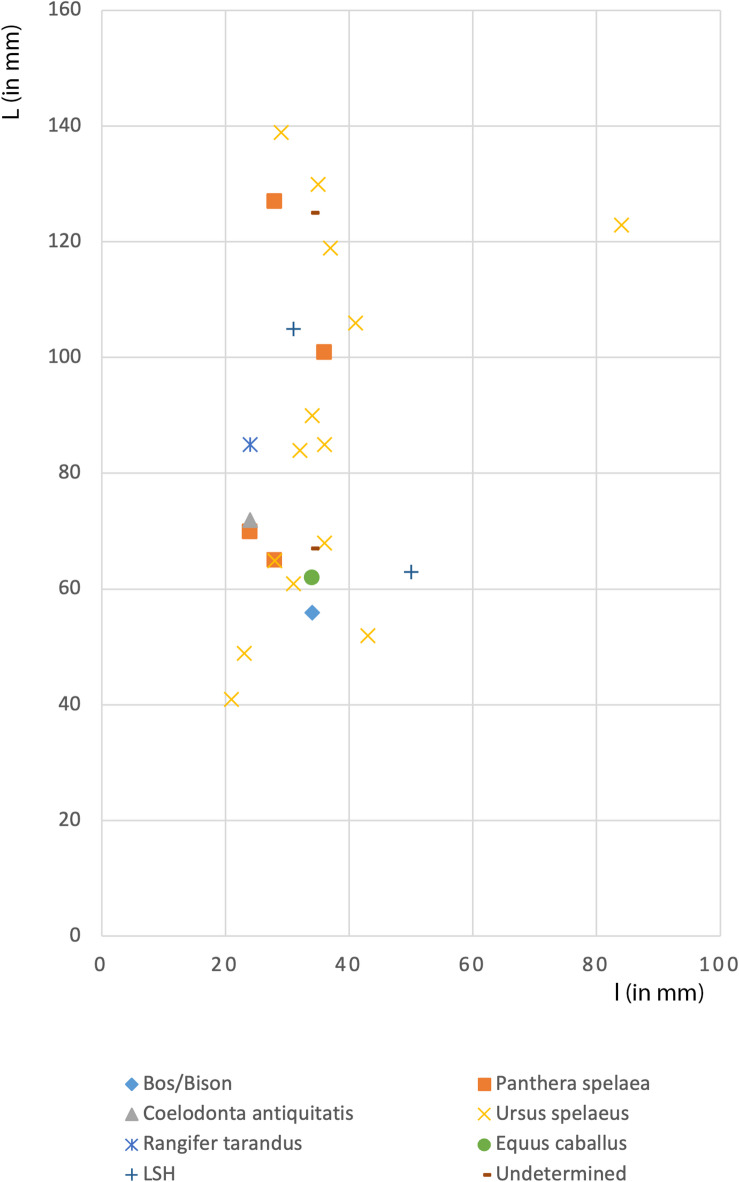
The size distribution of the bone retouchers from Scladina Unit 5 does not exhibit major differences between the species used.

In addition to the variety of species used, Neanderthals selected a range of anatomical elements to craft their tools, including fragments of metapodials, ribs, femurs, and tibias. These elements share a common characteristic: they were relatively fresh at the time of shaping and use. While it is possible that the mechanical properties of bones vary depending on the anatomical element and/or species selected, a comparison of the dimensions of the different tools (Fig. 6) reveals no significant size differences between the species used. Despite taxonomic variability—ranging from *Ursus spelaeus* to *Panthera spelaea*, *Bos/Bison*, and *Rangifer tarandus*—the tools exhibit a consistent dimensional range. The consistent size range across different taxa suggests that Neanderthals prioritized the size and shape of the blank over the species from which it derived. This degree of dimensional standardization implies a functional criterion in raw material selection, likely aimed at optimizing tool ergonomics and efficiency. Thus, the selection process reflects deliberate choices rooted in practical concerns rather than species-specific preferences, highlighting a sophisticated understanding of material properties and tool design.

### Species identification

Sc1986-1278-160 was originally thought to have been made from a cave bear tibia, based on morphological approach, and was published as such^23,35^. However, the popliteal line appeared to be very prominent, raising doubts about its identification. As with other bone tools (Table 2), it was decided to carry out a series of additional analyses designed to objectify the identifications (ZooMS and LC–MS/MS).

#### Zooarchaeology by mass spectrometry (ZooMS)

ZooMS analysis of the retouchers shows that they were made from *Ursus*, *Panthera*, *Panthera/Crocuta*, *Rangifer*, *Bos*/*Bison* and *Stephanorhinus* or *Coelodonta* bones. The Table 2 shows the list of retouchers and the ZooMS identification and the Supp. File S1 contains the ZooMS results. The identifications were based on database form Sam Presslee in University of York (https://docs.google.com/spreadsheets/d/1ipm9fFFyha8IEzRO2F5zVXIk0ldwYiWgX5pGqETzBco/edit?gid=1005946405#gid=1005946405). The distinction between Panthera and Hyaenidae was achieved using the peptide COL1A2T41 (E)^36^. For Panthera the mass of the peptide E was *m/z* 2820.354726 and *m/z* 2792.32343 for Hyaenidae. Only the identification of the retoucher Sc1986-1270-203 is ambiguous, and it is identified as either Panthera or Hyaenidae. The averages of deamidation values are 51.6% + /- 7.4% and 53.1% + /- 7.3% for bone fraction and acid fraction, respectively. These high deamidation values indicate that they are old proteins and correlate with the deamidation value for other analysis by paleoproteomics on Scladina cave bones^37^^.^ To ensure identification of the retouchers identified as *Panthera* and those with *Panthera*/*Crocuta* ambiguity, LC–MS/MS analyses were carried out.

#### Liquid chromatography–mass spectrometry (LC–MS/MS)

The samples number Sc1982-348-25, Sc1983-338-17, Sc1986-1270-203 and Sc1986-1278-160 have been analyzed by LC–MS/MS to validate the ZooMS results (Supp. File S2). *Panthera leo* type 1 collagen was identified with the highest score when the database containing COL1A1 and COL1A2 (1624 sequences) was queried. Single Amino acid variations at position 1071 have been identified on COL1A2 sequences for all samples. These variations have been identified on *Panthera spelaea* bones by proteomics in Bray and collaborators^37^. These results were confirmed using the ClassiCOL software, which identified the Panthera genus for all 4 samples (Supp. File S3, Figures S1–S4). PEAKSX analysis of sample Sc1983-382-120 identified the retoucher as Bos/Bison. The COL1A1 and COL1A2 proteins are identical between *Bos taurus* and *Bison bison* or *Bison bonasus*. However, COL3A1 contains SAPs (single amino polymophisms) and the GEPGAPGLK sequencing peptide (positions 120–128) is specific to *Bos taurus* COL3A1 (Supp. File S3, Figures S5–6). this identification has been validated with ClassiCOL software (Supp. File S3, Figure S7).

### Discussion and conclusions

The relationship between humans and large felids has been complex and multifaceted throughout history. In many cultures, lions, for instance, are both revered and feared: they are admired for their strength and majesty but are also considered threats to livestock and human safety. This ambivalence endures through time and space^38^. In the Upper Paleolithic, lions held profound symbolic value, as evidenced by iconic artworks like the Chauvet Cave paintings (France) or the *Löwenmensch* figurine from Hohlenstein-Stadel (Germany). Yet such symbolic associations are difficult to attribute to Neanderthal contexts, where evidence remains sparse and primarily functional. At Scladina Cave, the deliberate transformation of cave lion bones into tools reveals a markedly different dynamic—one grounded not in symbolism but in pragmatism and adaptive behavior.

The cave lion is primarily represented by limb bone fragments (Table 3), and the remains uncovered thus far do not suggest the presence of more than a single adult individual. Aside from the elements used as tools, no clear anthropogenic modifications are visible on the other preserved bones. However, the refitting of the tibia fragments emphasizes the intentional nature of the process—going beyond simple butchery waste recovery—and reflects the care invested in tool production. Alongside retouchers made from a cave bear femur, this case exemplifies the execution of a structured operational sequence (chaîne opératoire), like those commonly observed in lithic industries.

**Table 3:** List of the cave lion remains recovered within the Archeological Assemblage 5.

Accession number	Location	Layer	Anatomic portion	Side
Sc1982-347-1	G9	Vb	Hamate bone	L
Sc1982-348-8	G10	Vb	Scaphoid bone	L
Sc1982-348-25*	G10	Vb	Tibia shaft frag	L
Sc1982-346-2	F10	Vb	P^4^	
Sc1984-656-83	F11	5	Phalange 2	
Sc1983-273-62	F12	5	Phalange 3	
Sc1983-346-12	G12	5	Phalange 1	
Sc1983-407-92	E12	5	Patella	R
Sc1983-407-93	E12	5	Metatarsal III	R
Sc1983-428-82	E13	5	Metacarpal III	L
Sc1986-1270-203*	H13	5	Tibia shaft frag	L
Sc1983-186-21	F13	5	Scaphoid bone	R
Sc1983-236-52	E13	5	Calcaneus	R
Sc1984-717-184	E13	5	Metatarsal V	R
Sc1983-338-17*	D15	5	Tibia shaft frag	L
Sc1984-649-35	E16	5	Humerus shaft frag	L
Sc1986-1278-160*	H16	5	Tibia shaft frag	L
Sc1984-655-399	G17	5	Calcaneus frag	L
Sc1983-189-59	F19	5	Great sesamoid	
Sc1986-1289-245	H20	5	Tibia distal frag	L
Sc1985-921-318	F20	5	Lower canine	R
Sc1985-970-132	H21	5	3^rd^ Cuneiform	L
Sc2002-861-5	I23	5	Fibula	
Sc2001-120-127	E34	5	Cuneiform	
Sc2003-316-172	D37	5	Phalange 2	
Sc2003-316-168	D37	5	Hemimandible	
Sc1982-285-17	F5	Vb	Phalange 3	

The “*” following the accession number highlights the bone tools.

The selection of long bones for crafting bone tools aligns with broader Neanderthal technological behavior, as these elements were typically preferred across sites and species^24,39,40^. The findings from Unit 5 thus represent the earliest known evidence of bone tools made from cave lion remains, with the tibia-based artifacts demonstrating deliberate shaping, fracturing, and subsequent reuse as retouchers—further underscoring the complexity of Neanderthal technological practices.

Importantly, the morphometric analysis of all the retouchers found in Scladina Unit 5, spanning several species, reveals that Neanderthals employed a standardized approach to toolmaking. Despite the taxonomic diversity of the raw materials, including *Ursus spelaeus*, *Panthera spelaea*, *Bos* sp., *Rangifer tarandus*, and others, the dimensions of the retouchers remained consistent, indicating that size, shape, and perhaps structural integrity of the bone blank were the primary criteria for selection. This standardization suggests that Neanderthals were not opportunistically using any available bone but rather were deliberately selecting blanks, sometimes reshaping them, to meet specific functional requirements. The anatomical diversity of the selected bones (metapodials, ribs, femurs, tibias) and their consistent state of freshness at the time of shaping support this interpretation. In this light, the choice of species appears incidental to the practical utility of the material, reinforcing the idea of a function-driven technological tradition rather than one influenced by symbolism or cultural considerations. Moreover, the presence of woolly rhinoceros and reindeer further supports the hypothesis of site occupation during a relatively cold period, consistent with the end of the Saalian (MIS 6), as also indicated by recent chronological data^9^.

The deliberate exploitation of carnivore remains by Neanderthals at Scladina Cave is well-documented, particularly through anthropogenic modifications observed on cave bear bones^23^. While the abundance of bear remains suggests that Neanderthals may have hunted or scavenged animals that died naturally within the cave, the origin of the cave lion remains is more ambiguous. The bones of this individual show no evidence of natural alterations—such as erosion, weathering, or trampling—nor of carnivore activity. In addition, anthropogenic modifications including fractures, percussion notches, and cut marks indicate that the carcass was relatively fresh at the time of processing, a conclusion supported by the successful refitting of bone fragments. This raises the possibility that the lion was either hunted or recovered shortly after death, potentially reflecting a confrontation between Neanderthals and one of their main ecological competitors.

Although the possibility of opportunistic scavenging from a naturally deceased animal cannot be entirely excluded, the strategic use of the lion remains and the absence of taphonomic alterations strongly support a more active role. Given the extent of the excavations at Scladina, the meticulous recovery methods, and the spatial distribution of the lion bones—primarily concentrated in the first chamber of the cave, which is nearly completely excavated—the most plausible scenario is that Neanderthals deliberately brought a partial carcass into the site for processing. Whether this reflects hunting, scavenging, or defensive confrontation remains unresolved. However, if hunting was indeed involved, the act would carry considerable symbolic weight due to the high risk posed by such a formidable predator, especially compared to more commonly hunted prey like cervids or chamois^16^. These findings underscore Neanderthal behavioral flexibility and their capacity to exploit even dangerous carnivores for both functional and possibly symbolic purposes.

Ultimately, the discoveries at Scladina enrich our understanding of Neanderthal interaction with their environment and with other large predators. The integration of technological, zooarchaeological, and biomolecular data reveals not only the practical exploitation of carnivores but also a refined capacity for planning, selection, and reuse. These behaviors attest to Neanderthals’ advanced cognitive abilities and underscore their place as innovative, adaptive hominins capable of navigating complex ecological relationships.

The discovery at Scladina of bone retouchers crafted from cave lion remains represents an extraordinary and unparalleled finding within the Paleolithic archaeological record. It not only demonstrates the Neanderthals’ capacity to selectively exploit available resources, including those derived from large carnivores, but also reflects their ability to transform such remains into multifunctional tools following a structured operational sequence. The use of a predator as iconic and potentially dangerous as the cave lion—whether through active hunting or opportunistic recovery—reveals a high degree of behavioral flexibility and a sophisticated understanding of the technical properties of osseous materials. Beyond their rarity, these artifacts prompt a reevaluation of Neanderthal interactions with large carnivores, emphasizing that these animals were not merely ecological competitors but could also serve practical and possibly symbolic purposes in Neanderthal lifeways.

## Methods

The material unearthed in Unit 5 underwent a systematic review aimed at identifying potential bone retouchers. This fruitful analysis documented a relatively intense exploitation of cave bears, although other species were also identified but are less well represented^23,24,41^. This study notably proposed a genuine debitage sequence on a cave bear femur, demonstrating both access to a relatively fresh carcass and a series of reasoned actions to extract several supports from the diaphysis transformed into tools^23^. Although refitting and morphological studies identified numerous species, several tools could not be identified at the specific level due to the significant modifications applied by Neanderthals. Consequently, we resorted to proteomic analyses (Table 2), which had previously been tested and yielded conclusive results^37^.

The cave lion remains excavated so far seem to belong to a single left tibia of an adult specimen, according to the degree of ossification and fusion of the epiphyses. The individual is mainly represented by limb bones (Table 3).

**Fig. 7:**
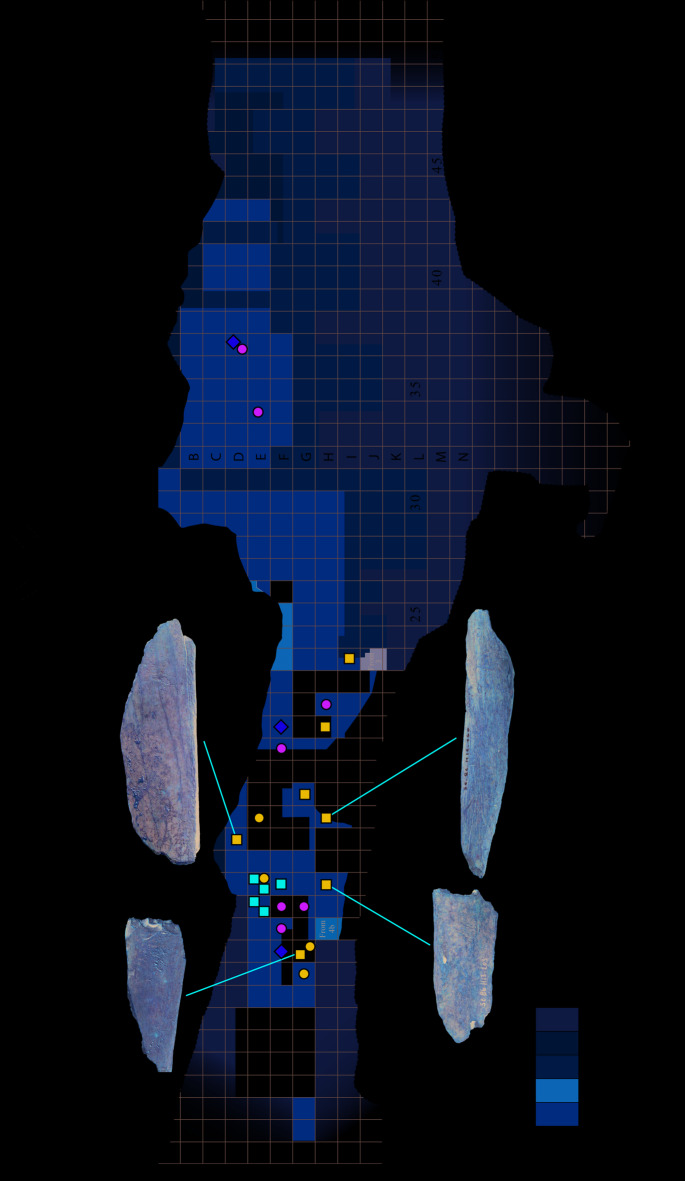
Spatial distribution of the cave lion remains (Table 2). Cranial fragments are figured by yellow diamonds, left lower limb by blue squares, right lower limb by red squares, right upper limb in blue dots, and non-lateralized bones by green dots.

Most of the cave lion remains are distributed in the first part of the cave, close to the current porch (Fig. 7). This concentration is the same as that already observed for tools made from cave bear remains^23^.

### Microwear analysis

The samples Sc1986-1278-160 and Sc1982-348-25 were subjected to preliminary microwear analysis. The surface and the edges of the tool were examined and photographed with a Zeiss AxioZoom V16 macroscope with magnification range between 7-112x. Zeiss ZEN Core software (v.3.4; https://www.zeiss.com/microscopy/en/products/software/zen-core.html) was used to acquire the micrographs. Areas of interest were also assessed with an Olympus BX53M microscope under 50 ×, 100 ×, and 200 × magnifications. Micrographs were taken with the use of a Nikon D750 DSLR camera and Best Scientific 1.9X coupler and captured with Helicon Remote software (v.4.5.3; https://www.heliconsoft.com/) and processed with Helicon Focus software (v.8.3.4; https://www.heliconsoft.com/).

### Nitrogen content analysis

A small fragment of the bone retoucher Sc1983-338-17 has been submitted to a nitrogen content analysis to evaluate the possibility to extract collagen. This extraction attempt was made to perform carbon and nitrogen isotopic analyses and compare them to cave bears and cave lions’ data from Scladina Cave. Following the methodology presented by Bocherens and colleagues^42^, 5 mg of bone powder were analyzed using an Elemental Analyzer to measure its nitrogen content, a proxy for collagen in ancient bone. The obtained results (%N = 0.23%) are below the threshold of 0.4%, equivalent to 10% of the original collagen and therefore indicate that the collagen content is insufficient to yield enough collagen for isotopic analysis. No further analysis was attempted on this specimen.

### Proteins digestion on plate

Bone proteins were analyzed using methods by Bray and collaborators^43^. All solutions were eluted through the wells using a vacuum manifold (Merck KGaA, Darmstadt, Germany) pumped by a DS 102 rotary vane pump (Agilent, Santa Clara, USA). The wells of a 0.45 μm hydrophobic-high protein binding (MSIPS4510, Millipore, Billerica, MA, USA) were prepared by washing with 70% ethanol flowed through the filter. In each well, 1–5 mg of bone powder was deposited and 100 µL of demineralization solution (5% v/v TFA (trifluoroacetic acid)) were added. The plate was incubated at 4 °C for 16 h without shaking. The demineralization solution in TFA was recovered in a V-bottom well-collecting plate by applying vacuum. Then 6 µL of NaOH 6 M were added to neutralize the TFA and 100 µL of 100 mM ABC (ammonium bicarbonate) pH 8.8. The plate was kept at 4 °C for digestion. The bone powder in each well was washed three times with 100 µL of digestion buffer (50 mM ABC, pH 8.8). The plate was incubated at 65 °C for 1 h on a heating stirrer MHR23 (Hettich, Tuttlingen, Germany) for collagen gelatinization. The digestion of bone powder and demineralization solution was performed by adding 0.5 μg of sequencing grade trypsin (Promega, Madison, WI, USA) and incubating the mixture at 37 °C for 16 h with shaking on a heating stirrer. Peptides from bone powder were recovered in a V-bottom well-collecting plate by applying vacuum and the plate was washed one time with digestion buffer. Tryptic peptides from bone powder and demineralization solution were acidified with 1 µL of acetic acid (final concentration 0.5% of acetic acid). Tryptic peptides were desalted on 96 wells plates C18 (Affinisep, Petit-Couronne, France). Before analysis, each sample was resuspended in 10 µL of H_2_O, 0.1% formic acid. The concentration of peptide was measured with DS-11 + (Denovix, Wilmington, USA) at 214 nm.

### MALDI analysis

MALDI analyses were conducted using the protocol described by Bray and collaborators^43^. Desalted peptides (1 µL) were deposited on 384 Ground steel MALDI plates (Bruker Daltonics, Bremen, Germany), then 1 µL of HCCA matrix at 10 mg/mL in ACN/H_2_O 80:20 v/v 0.1% formic acid was added for each sample spot and dried at ambient temperature. MALDI-FTICR experiments were carried out on a Bruker 9.4 Tesla SolariX XR FTICR mass spectrometer (Bruker Daltonics, Bremen, Germany). A Bruker Smartbeam-II Laser System was used for irradiation at a frequency of 1000 Hz and using the “Minimum” predefined shot pattern. MALDI-FTICR spectra were generated from 500 laser shots in the *m/z* range from 693.01 to 5000 with 2 M data points (i.e., transient length of 5.0332 s). Twenty spectra were averaged. The transfer time of the ICR cell was set to 1.2 ms and the quadrupole mass filter operating in RF-only mode was set at *m/z* 600.

### LC–MS/MS analysis

LC–MS/MS analyses were performed on an Orbitrap Q Exactive plus mass spectrometer hyphenated to a U3000 RSLC Microfluidic HPLC System (ThermoFisher Scientific, Waltham, Massachusetts, USA) according to the method presented in Bray and collaborators^43^. 1 μl of the peptide mixture at a concentration of 1 µg/µL was injected with solvent A (5% acetonitrile and 0.1% formic acid v/v) for 3 min at a flow rate of 10 μl.min^−1^ on an Acclaim PepMap100 C18 pre-column (5 μm, 300 μm i.d. × 5 mm) from ThermoFisher Scientific. The peptides were then separated on a C18 Acclaim PepMap100 C18 reversed phase column (3 μm, 75 µm i.d. × 500 mm), using a linear gradient (5–40%) of solution B (75% acetonitrile and 0.1% formic acid) at a rate of 250 nL.min^−1^ in 165 min and then 100% of solution B in 5 min. The column was washed for 5 min with buffer B and then re-equilibrated with buffer A. The column and the pre-column were placed in an oven at a temperature of 45 °C. The total duration of the analysis was 185 min. The LC runs were acquired in positive ion mode with MS scans from *m/z* 350 to 1600 in the Orbitrap mass analyser at 70,000 resolution at *m/z* 400. The automatic gain control was set at 3E6. Sequentially MS/MS scans were acquired in the high-energy collision dissociation cell for the 15 most-intense ions detected in the full MS survey scan at 35,000 resolution at *m/z* 400. Automatic gain control was set at 5E5, and the normalized collision energy was set to 30 eV. Dynamic exclusion was set at 30 s and ions with 1 and more than 8 charges were excluded.

### Bioinformatics for MALDI data

MALDI FTICR were processed using Compass DataAnalysis (V.5.0.; https://bruker-compass-dataanalysis.software.informer.com) SNAP algorithm was employed with the following parameters of S/N > 3 and quality 0.6. The procedure for the deamidation value calculation from MALDI FTICR was based on Bray and colleagues^43^ and the identification of taxonomic rank was realized with data from the literature and ZooMS data base from Sam Presslee in University of York (https://docs.google.com/spreadsheets/d/1ipm9fFFyha8IEzRO2F5zVXIk0ldwYiWgX5pGqETzBco/edit?gid=1005946405#gid=1005946405).

### Bioinformatics for LC–MS/MS data

Proteomics data were processed with PEAKS X + (https://www.bioinfor.com/peaks-studio-x-plus) against a home-made database containing 1,765 collagen sequences extracted from NCBI database (All_Collagen, downloaded 08-2023) restricted to Mammalian, as already described by Bray and collaborators^43^. Precursor’s mass tolerance was fixed to 10 ppm and fragment ion mass tolerance to 0.02 Da. Semi-trypsin digestion mode was used. Cysteine carbamidomethylation was set as fixed modification. Methionine oxidation and asparagine, glutamine deamidation and hydroxylation of amino acids (RYFPNKD) were selected as variable modifications. PEAKS PTM and SPIDER ran with the same parameters. Results were filtered using the following criteria: protein score − 10logP ≥ 20, 1% peptide False Discovery Rate (FDR), PTM with Ascore = 20, mutation ion intensity = 5% and *Denovo* ALC ≥ 50%. Peptides with amino acids substitutions were filtered with minimal intensity set as 1E + 7.

A second bioinformatic analysis was done with Mascot against Mammalian database from NCBI containing 9,016,701 sequences. Peptide mass tolerance and fragment mass tolerance were fixed to 10 ppm. Semi-trypsin digestion mode was used. Cysteine carbamidomethylation was set as fixed modification. Proline oxidation and asparagine and glutamine deamidation were selected as variable modifications. CSV file were exported and analyzed with ClassiCOL (V.1.0.2; https://github.com/EngelsI/ClassiCOL/tree/main/ClassiCOL_version_1_0_2) with default parameters^44^.

### Energy-dispersive X-ray (EDX) spectroscopy

Scanning electron microscope (SEM) images were acquired using a Quanta 200 ESEM, manufactured by FEI. X-ray emissions associated with imaging were collected using an energy dispersive X-ray spectrometer (EDS APOLLO 10, Silicon Drift detector by EDAX), enabling semi-quantitative chemical analysis of elements ranging from beryllium (Z = 4) to uranium (Z = 92).

The imaging was conducted under low-vacuum conditions (15 Pa) of water vapor, with a 30 kV incident electron beam and a working distance of 13 mm. The SEM images were produced by detecting backscattered electrons (BSE), allowing the detection of different phases based on their molecular weights.

For chemical analysis, EDS data can be obtained either as individual data points or through chemical mapping. This combination of techniques offers a rapid, non-destructive method for analyzing and distriminating between lithic materials.

## Electronic supplementary material

Below is the link to the electronic supplementary material.

Supplementary Material 1

Supplementary Material 2

Supplementary Material 3

## Data Availability

The mass spectrometry proteomics data (MALDI-FTICR, LC–MS/MS raw data and PEAKS X + results) have been deposited on the ProteomeXchange Consortium (http://proteomecentral.proteomexchange.org) via the PRIDE partner repository47 with the data set identifier PXD055624.
