# Supplementary material for: Earliest evidence of Neanderthal multifunctional bone tool production from cave lion (Panthera spelaea) remains
Source: Sci Rep. 2025 Jul 5;15:24010. doi: 10.1038/s41598-025-08588-w (PMC12228739; doi:10.1038/s41598-025-08588-w)
Supplement: Supplementary file 3 — Supplementary Material 3 [file 41598_2025_8588_MOESM3_ESM.pptx]

## Slide 1
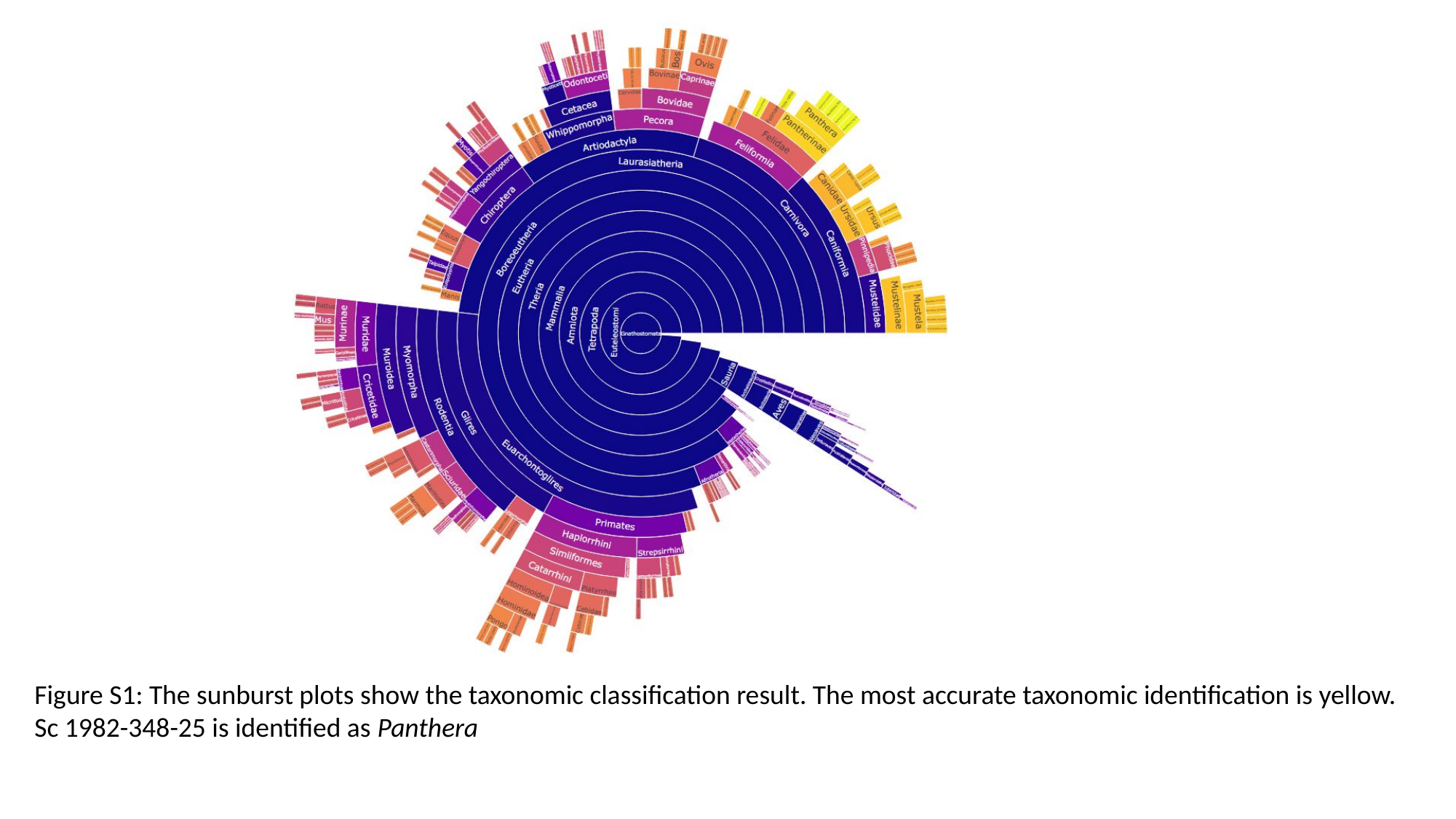

Figure S1: The sunburst plots show the taxonomic classification result. The most accurate taxonomic identification is yellow. Sc 1982-348-25 is identified as Panthera

## Slide 2
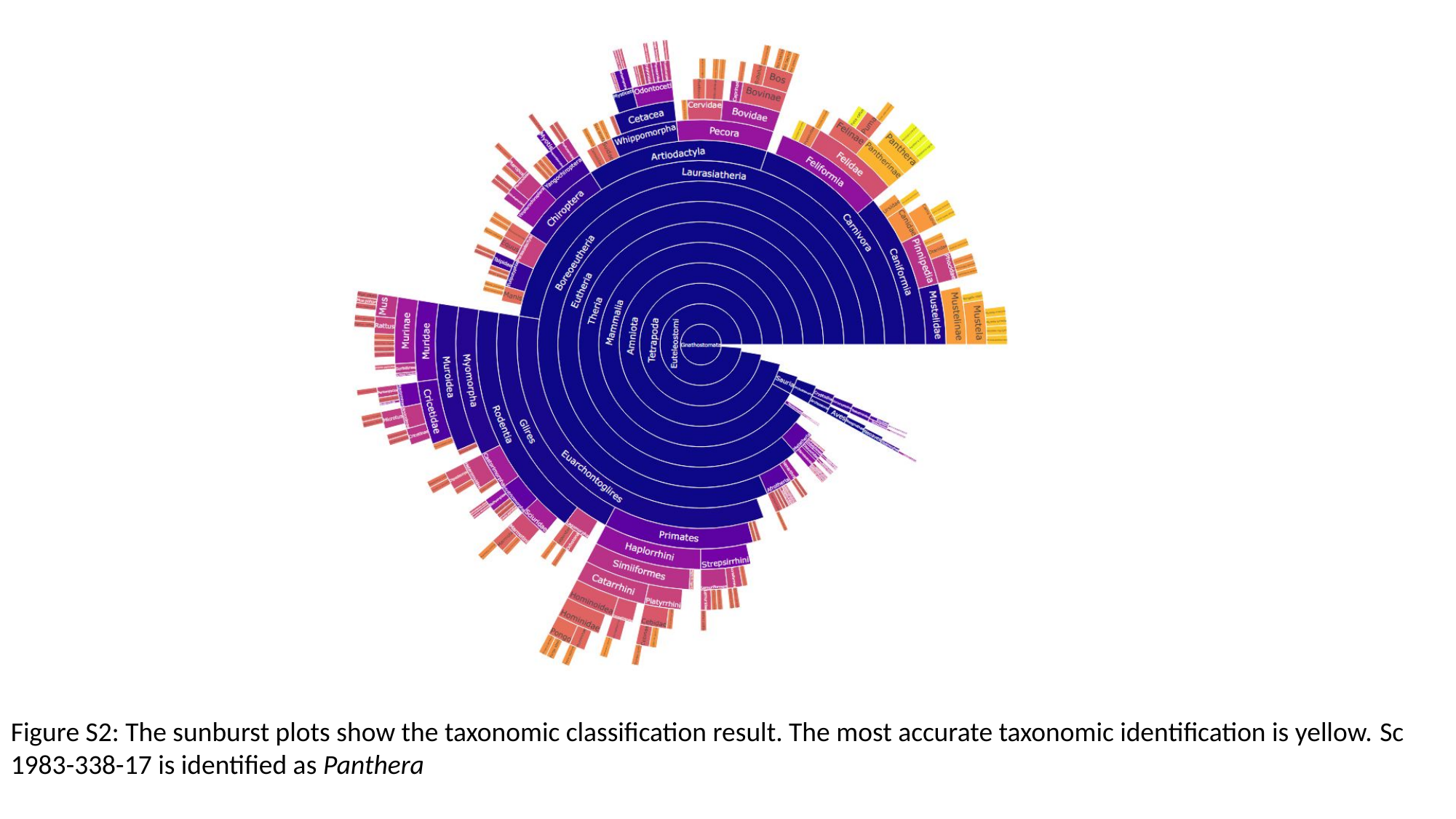

Figure S2: The sunburst plots show the taxonomic classification result. The most accurate taxonomic identification is yellow. Sc 1983-338-17 is identified as Panthera

## Slide 3
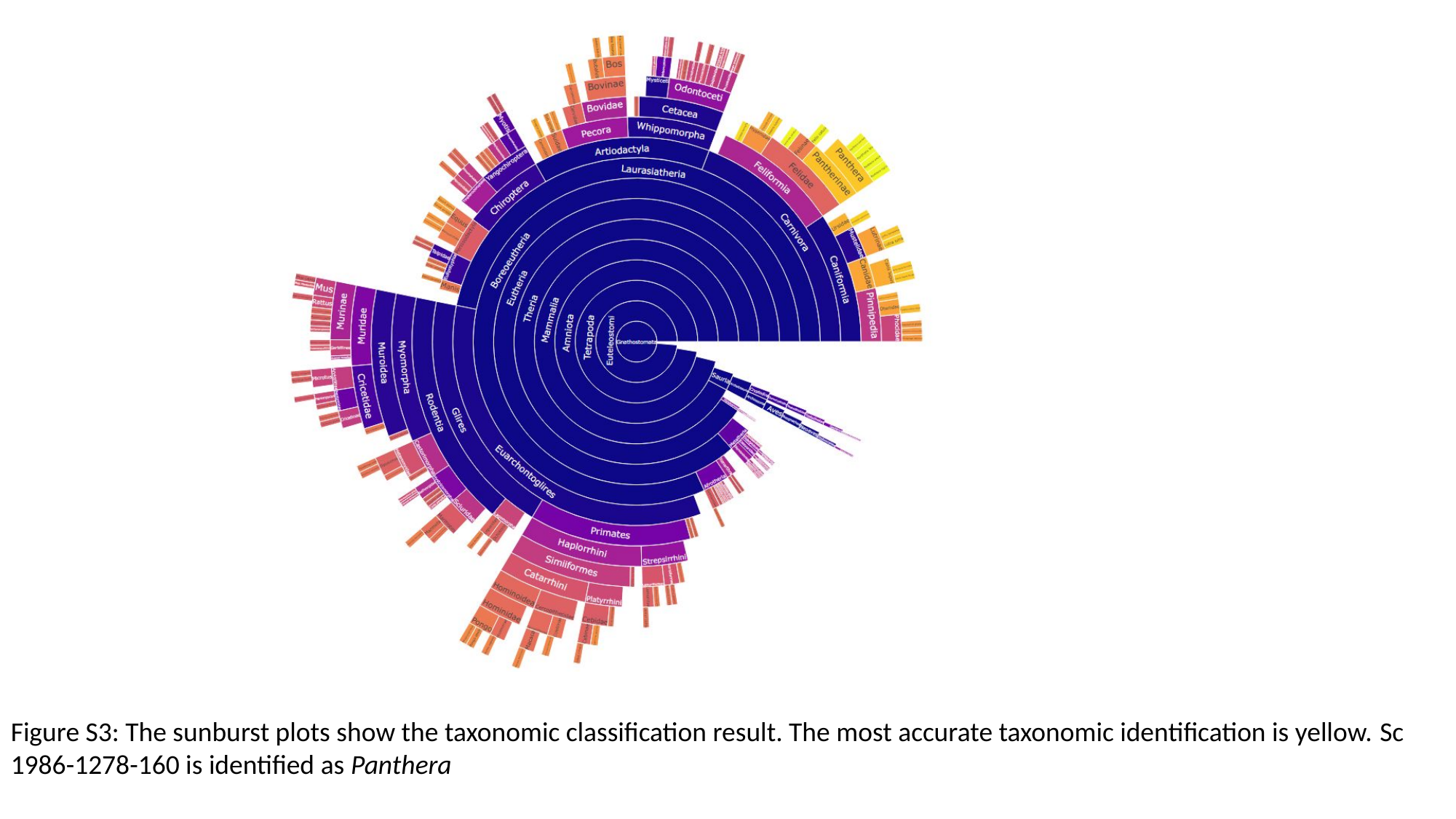

Figure S3: The sunburst plots show the taxonomic classification result. The most accurate taxonomic identification is yellow. Sc 1986-1278-160 is identified as Panthera

## Slide 4
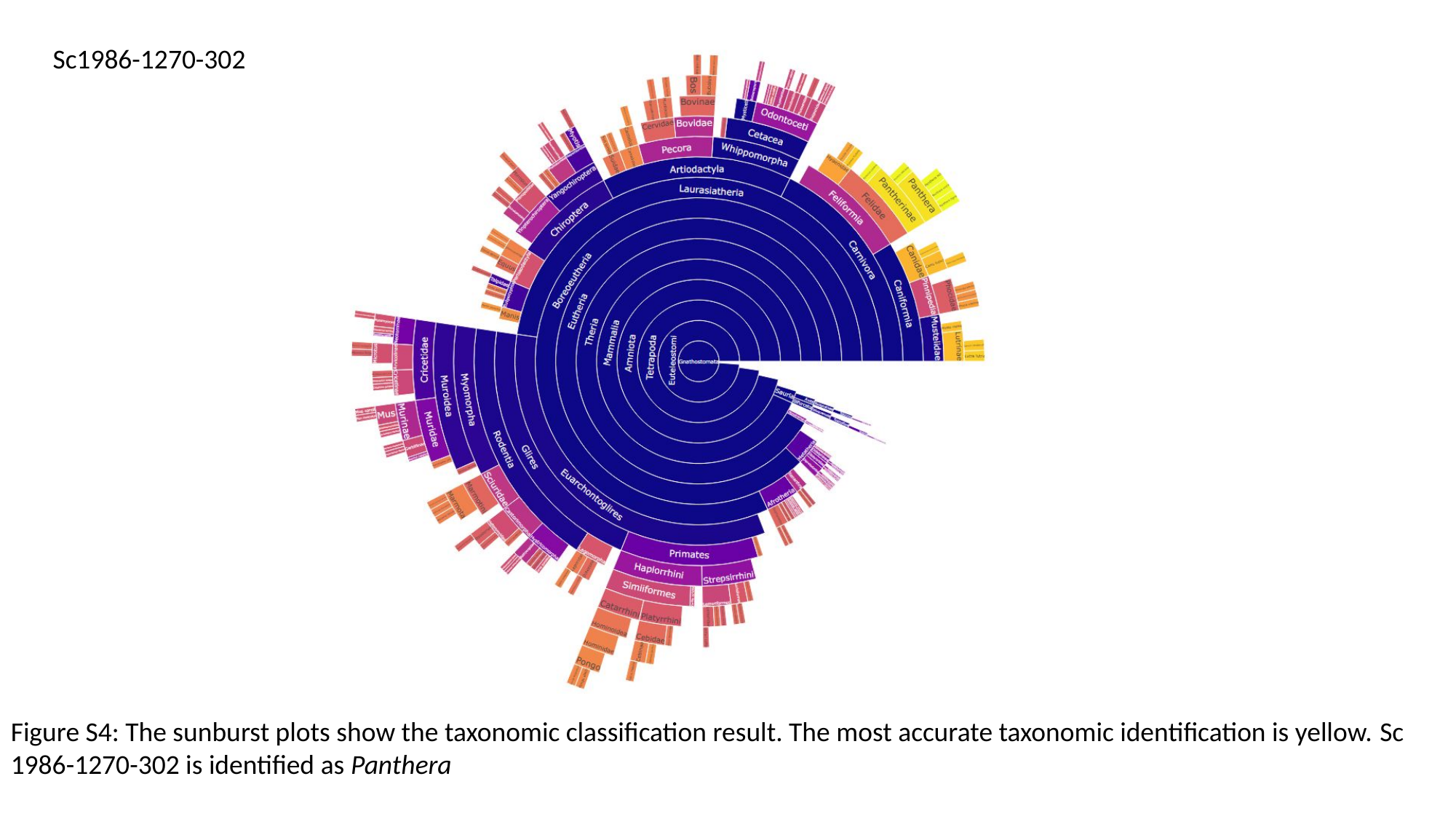

Sc1986-1270-302
Figure S4: The sunburst plots show the taxonomic classification result. The most accurate taxonomic identification is yellow. Sc 1986-1270-302 is identified as Panthera

## Slide 5
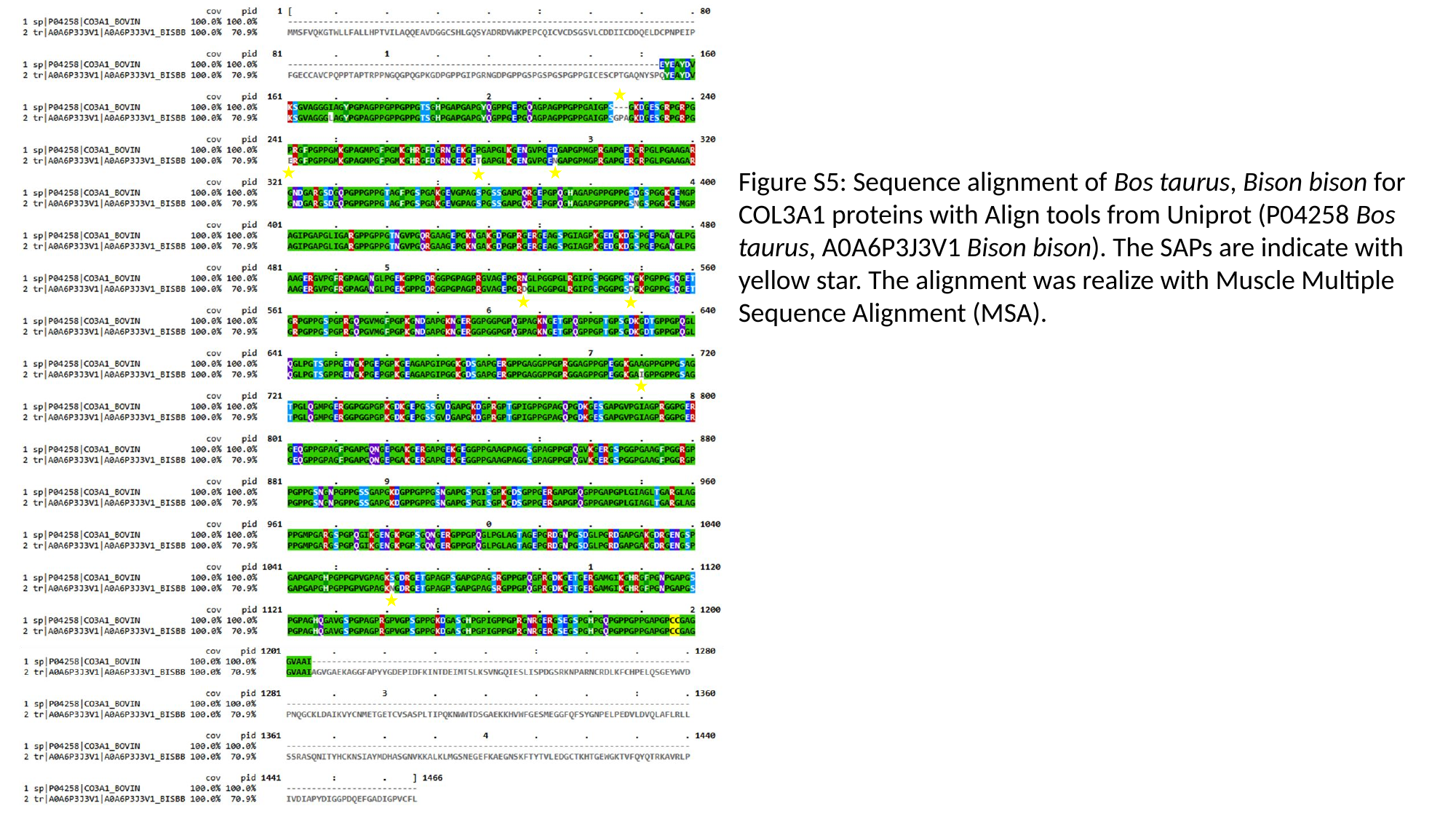

Figure S5: Sequence alignment of Bos taurus, Bison bison for COL3A1 proteins with Align tools from Uniprot (P04258 Bos taurus, A0A6P3J3V1 Bison bison). The SAPs are indicate with yellow star. The alignment was realize with Muscle Multiple Sequence Alignment (MSA).

## Slide 6
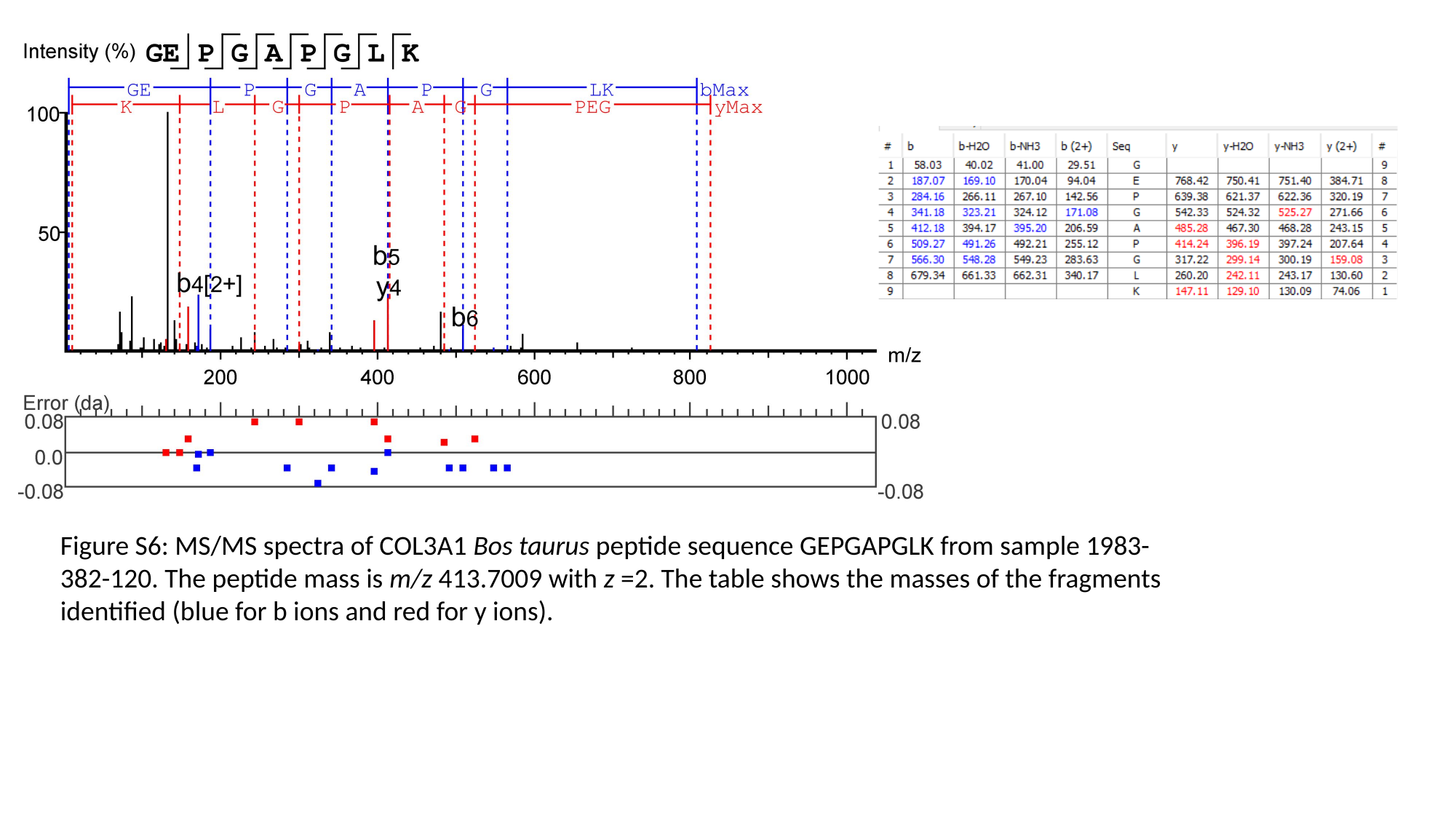

Figure S6: MS/MS spectra of COL3A1 Bos taurus peptide sequence GEPGAPGLK from sample 1983-382-120. The peptide mass is m/z 413.7009 with z =2. The table shows the masses of the fragments identified (blue for b ions and red for y ions).

## Slide 7
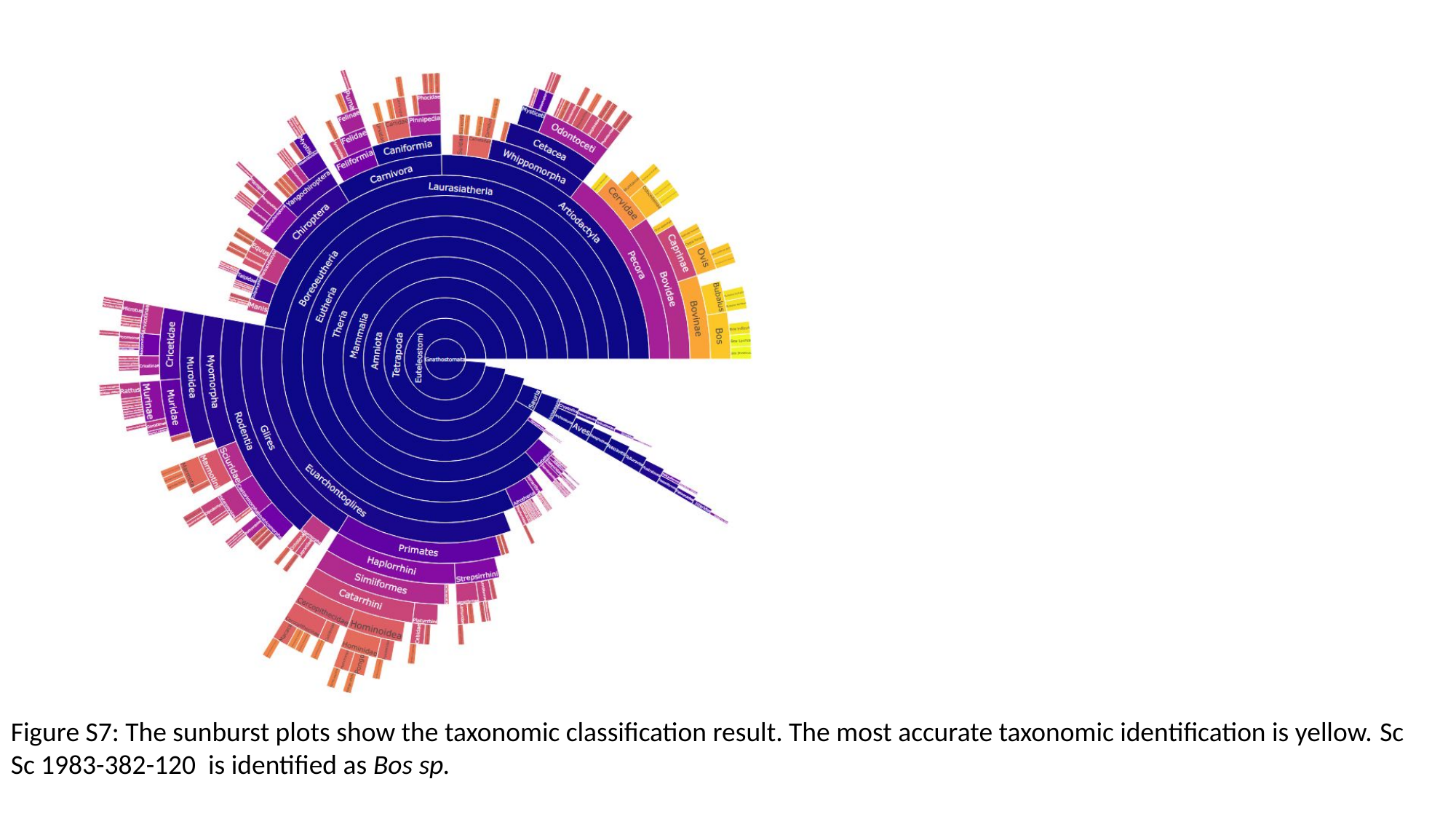

Figure S7: The sunburst plots show the taxonomic classification result. The most accurate taxonomic identification is yellow. Sc Sc 1983-382-120 is identified as Bos sp.
